# Emerging Molecular Determinants and Protective Strategies in Heart Disease: What’s New in the *Journal of Clinical Medicine*? Outlook to the Future

**DOI:** 10.3390/jcm12144564

**Published:** 2023-07-08

**Authors:** Carmine Rocca, Tommaso Angelone

**Affiliations:** 1 Cellular and Molecular Cardiovascular Pathophysiology Laboratory, Department of Biology, Ecology and Earth Sciences (DiBEST), University of Calabria, 87036 Rende, Italy; carmine.rocca@unical.it; 2 National Institute of Cardiovascular Research (INRC), 40126 Bologna, Italy

Cardiovascular diseases (CVD), including coronary heart disease (CHD), heart attacks, stroke, heart failure (HF), and peripheral artery disease, still represent the leading cause of death globally, taking an estimated 17.9 million lives each year. However, mortality from CVD has significantly decreased in recent decades, particularly thanks to advances in cardiovascular basic and translational research and clinical progress; this has led to the development of drugs targeting selective pathways and other effective cardioprotective strategies, resulting in improved clinical outcomes [1]. Therefore, it is necessary to consider, in the future, cardiovascular research as an essential service to further advance the prevention and treatment of CVD. In particular, improving our understanding of the cellular and molecular mechanisms and genetic factors behind the pathophysiological processes driving CVD is fundamental in identifying novel molecular determinants and cardioprotective strategies that are able, for example, to limit the infarct size following an acute myocardial infarction (AMI), improve left ventricular (LV) function and reduce adverse LV remodeling, and reduce or prevent the cardiovascular side effects of cancer therapy. In this scenario, promising progress has been achieved and published recently in the *Journal of Clinical Medicine*.

CHD is the most common type of heart disease, the consequences of which are usually attributed to the detrimental effects induced by acute myocardial ischemia/reperfusion injury (MI/R), thereby increasing the risk of arrhythmias and HF. A novel reperfusion strategy for primary percutaneous coronary intervention (PCI), named “volume-controlled reperfusion (VCR)”, in patients with acute ST-segment elevation myocardial infarction (STEMI), was proposed in a pilot study involving 30 patients by He et al. (2023) [2]. VCR is based on an accurate intermittent slow distal reperfusion using a manual intra-aspiration catheter after suspending the antegrade flow using a prophylactic inflated balloon. According to the authors, the aim of VCR is to overcome the difficulties that may be associated with the classical post-conditioning maneuver, which is based on applying a sequence of MI/R episodes, which are induced by repeated cycles of balloon-catheter inflation and deflation immediately after reopening the occluded vessel. Despite the fact that this procedure has been shown to induce cardioprotection in several pre-clinical studies and clinical proof of concept trials, there have been disappointing results in large randomized controlled trials (such as POST, DANAMI 3 i-POST), in part due to the multiple pathways that reperfusion can contribute to cell death and to the possible additional damage to the coronary endothelium and myocardium, with the increased risk of thrombus dislodgment, following the repeated inflation and deflation of the balloon during post-conditioning. Although VCR needs to be further studied, this approach possesses promising therapeutic potential in the context of STEMI patient-specific treatment. In this regard, an insightful review [3] recently discussed the pathophysiological complexity of MI/R and microcirculation injuries leading to myocardial dysfunctions, providing possible explanations behind the conflicting results related to pharmacological and mechanical cardioprotective interventions between pre-clinical MI/R reports and studies in patients with STEMI. The authors also proposed a “multitarget therapeutic approach” to prevent post-STEMI LV remodeling based on emerging data, indicating that combining additive or synergistic multitarget therapies could be a more effective strategy in affording optimal cardioprotection [4].

Interesting evidence has indicated a significant relationship between specific endogenous factors and AMI. In particular, in a prospective cohort study of STEMI patients who underwent coronary artery angiography, the authors suggested that higher levels of the B cell activating factor (BAFF), a member of the tumor necrosis factor (TNF) superfamily with a significant role in MI/R and atherosclerosis lesions and progression, could represent, in the acute phase, an independent predictor of the incidence of major adverse cardiovascular events in the post-MI period [5]. On the other hand, Kożuch et al. (2022) [6] indicated, in a prospective study, the correlation between growth differentiation factor 15 (GDF-15), as a member of the transforming growth factor superfamily involved in apoptotic and inflammatory pathway regulation, and disturbances in the microcirculatory reflow in patients with STEMI after PCI. Similar results were obtained in a prospective, observational, open-label trial involving 267 STEMI patients treated with PCI [7]. Here, high levels of neutrophil gelatinase-associated lipocalin (NGAL), a protein stored in both neutrophil blood cells and renal tubular cells that act as an early and sensitive marker for acute kidney injury and reflect endothelial dysfunction and plaque instability, have been shown to associate with major adverse renal and cardiovascular complications, which are independent of classical inflammatory markers, including white blood cell (WBC) counts and C-reactive proteins (CRP). Additional investigations have proved the real biomarker role of BAFF, GDF-15, and NGAL that is required; however, these encouraging results suggest that these emerging factors might represent additional prognostic markers of STEMI and MI/R injury with a possible implication in cardiovascular risk stratification.

On the other hand, recent mechanistic studies have significantly contributed to enhancing the knowledge surrounding pathophysiological determinants of specific heart diseases. This is the case of dilated cardiomyopathy (DCM), a complex myocardial disorder representing a major cause of HF and death, where genetic components play an important role in its pathogenesis, accounting for at least one-third of cases of “idiopathic” DCM. A study recruiting 62 patients presenting with idiopathic DCM elucidated a complex series of events, linking alterations of proteostasis with inflammation, where numerous molecular actors, including miR-22, the phosphatase PP2Cm, branched-chain amino acids, mTOR, and an autophagy lysosomal pathway, could participate in DCM pathogenesis and may serve as therapeutic targets for myocardial disease [8].

Clinical and experimental evidence indicates that excessive aldosterone signaling could negatively affect cardiovascular function; indeed, inhibiting aldosterone receptors has been shown to mitigate LV hypertrophy and decrease cardiovascular-related mortality. A recent study indicated, in human failing hearts independent of etiology, a strict relationship between the high myocardial expression of aldosterone receptor and aquaporin-1 and intracellular water overloading and cardiomyocyte swelling, which can culminate in cardiac dysfunction, providing a key cellular and morpho-molecular basis underlying aldosterone-dependent human HF progression [9].

Extensive evidence has shown that another important health problem refers to cardiotoxicity arising from cancer treatments. Pre-clinical and clinical data demonstrated that chemotherapy, radiotherapy, and cancer itself could induce functional or structural heart damage, affecting life expectancy amongst cancer survivors, thus representing a relevant issue in clinical practice. Accordingly, experimental and clinical cardio-oncology has emerged as a key area aimed at studying, identifying, and treating cardiovascular complications following cancer therapy (e.g., cytotoxic agents, immunotherapies, radiation, and hormone therapies). Cardiotoxicity can manifest as an early or late event after therapy and may vary from subclinical myocardial dysfunctions to irreversible HF. Numerous pharmacological cardioprotective interventions, including angiotensin-converting enzyme (ACE) inhibitors, angiotensin receptor blockers, and beta-blockers, could be combined with cancer regimens in clinics. However, at the moment, these drugs may not be used for all patients since additional population evidence is required to determine their efficacy and optimal dosage. Therefore, the early identification of cancer therapy-related cardiovascular toxicity remains a critical issue in adequately managing CVD caused directly or indirectly by cancer. Echocardiography, in combination with relative changes in the global longitudinal strain (GLS) and levels of serum cardiac biomarkers (i.e., cardiac troponins), is recommended in the cardio-oncology guidelines to monitor cardiac functions and detect early changes in the LV function during anticancer therapy, as well as the entire management of cardiotoxicity. In this context, recently, a single-center observational study proposed serial myocardial work measurements through a non-invasive LV pressure-strain loop for patient risk stratification and the early detection of cardiotoxicity following chemotherapy (anthracycline therapy w/wo trastuzumab) in 50 breast cancer patients that were monitored before and 3, 6, and 12 months after the initiation of chemotherapy. The authors investigated temporal changes and longitudinal trajectories in myocardial work indices during cancer treatment, providing evidence of the clinical effectiveness of serial myocardial work analysis as an additional confirmatory marker. Interestingly, non-invasive myocardial work indices derived from LV pressure–strain loops could overcome the load-dependent limitations of GLS, which was affected by blood pressure changes. Therefore, this study further contributed to validating this method in cancer patients exposed to chemotherapeutics, for which the available evidence is still limited [10].

Together with additional approaches for the early detection of cardiotoxicity, strong evidence derived from current guidelines indicates how additional cardiac-specific biomarkers and more efficient cardioprotective strategies are crucial for the early detection and treatment of CVD in cancer patients, respectively. In particular, Mauro and collaborators (2023) [11] recently published a pragmatic multistep approach based on the expert opinion of a multidisciplinary team and on the current guidelines for the management of breast cancer patients during treatment with anthracyclines and/or human epidermal growth factor receptor 2 inhibitors, highlighting the importance of (i) validating the surveillance protocols for anticancer drugs based on current guidelines and recommendations; (ii) optimal strategies to manage cardiovascular side effects; and (iii) a long-term surveillance strategy for high- and very high-risk patients. On the other hand, Gioffré and colleagues [12] performed a proof-of-concept clinical study testing the potential role of specific circulating miRNAs as early predictors of cardiac troponins elevation in 88 breast cancer patients upon treatment with anthracyclines. It was well established that cardiac troponin represented the most commonly adopted biomarker for monitoring the onset of anthracycline-induced cardiotoxicity. Although cardiac troponins possess a good negative predictive value, they have sub-optimal positive predictive potential since their increase during anthracycline administration can indicate an increased propensity to develop cardiac dysfunction, but without indicating the timing of its onset. Therefore, additional biomarkers with high specificity and accuracy to be coupled with cardiac troponins for the early assessment of cardiotoxicity are required. The authors identified a serum signature represented by specific miRNAs (i.e., miR-122-5p, miR-499-5p, and miR-885-5p), which can predict adverse cardiac responses to the anthracycline doxorubicin before its administration. Despite the small sample size of the cohorts, this study shows the promising potential of miRNAs in contributing to the selection of the least cardiac-harmful treatment for patients and/or identifying high-risk patients for treatment with cardio-preventive treatment before chemotherapy.

Looking ahead, there is a need to adhere to the conventional guidelines for managing CVD while implementing basic, translational, and clinical cardiovascular research as a fundamental step for identifying the specific causes of CVD and the set-up of precision therapies.
